# Development of a two-dimensional liquid chromatography-tandem mass-spectrometry method for the determination of vitamin D2 in mushrooms

**DOI:** 10.1007/s00216-022-04281-3

**Published:** 2022-08-19

**Authors:** Sieglinde Zelzer, Andreas Meinitzer, Dietmar Enko, Markus Herrmann, Walter Goessler

**Affiliations:** 1 Clinical Institute of Medical and Chemical Laboratory Diagnostics, Medical University of Graz, Graz, Austria; 2 Institute of Chemistry, University of Graz, Graz, Austria; 3 Institute of Clinical Chemistry and Laboratory Medicine, General Hospital Hochsteiermark, Leoben, Austria

**Keywords:** Vitamin D2, LC–MS/MS, Mushrooms, Standard addition, Irradiation

## Abstract

Different foods, especially mushrooms, are a valuable source of vitamin D2. However, published concentrations in mushrooms show large variabilities. One reason for this is certainly the high biological variability caused by growth conditions, and another could also be found in the analytical methodology. Therefore, this study aimed to develop a sensitive and highly selective two-dimensional liquid chromatography mass spectrometry (LC–MS/MS) method for vitamin D2 analysis in mushrooms. After validation, the method was applied to four different mushroom species. The developed method with a one-step extraction procedure showed a limit of detection of 0.01 µg vitamin D2/g dry mass (DM), a limit of quantification of 0.05 µg vitamin D2/g DM, and recovery rates between 87.6 and 94.8%. The total run time including the re-equilibration of the columns for the next injection was 7.5 min. After adding increased concentrations of pure substance to *Pleurotus ostreatus*,* Lentinula edodes*, and *brown* and *white button mushrooms* the standard addition plot showed excellent correlation coefficients (*R*^2^) of > 0.9994. Mean vitamin D2 concentrations were observed at 0.122 ± 0.007, 0.074 ± 0.005, 0.099 ± 0.007, and 0.073 ± 0.005 µg/g DM. The coefficient of variation (CV) was between 5.1 and 7.6%. This well-optimized, sensitive LC–MS/MS method, with a fast and simple sample preparation and a short run time, can be applied to future studies especially in different mushroom species with variable growing conditions. This will improve our knowledge about the vitamin D2 content in mushrooms.

**Graphical abstract:**

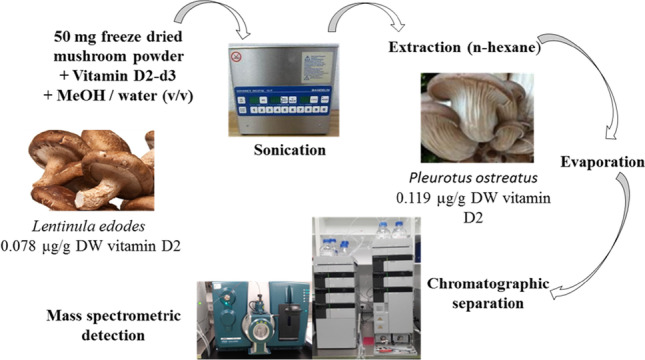

**Supplementary Information:**

The online version contains supplementary material available at 10.1007/s00216-022-04281-3.

## Introduction

Vitamin D deficiency is a worldwide common condition, which is assessed with 25 hydroxyvitamin D (25(OH)D) blood concentrations. Independently of the 25(OH)D measurement methodology, a very large number of children and adults suffer from it [1]. Many studies have suggested that an inadequate intake of vitamin D is associated with different pathophysiological processes. This fact may increase the risk of osteomalacia, rickets and several other diseases including psoriasis, multiple sclerosis, inflammatory bowel disease, type 1 and 2 diabetes, hypertension, cardiovascular disease, metabolic syndrome, and various forms of cancer [2–4]. A general public health intervention, which includes vitamin D supplementation and systemic vitamin D food fortification to avoid severe vitamin D deficiency, would appear to be important [5].

The recommended daily vitamin D intake is age-dependent, namely an intake of 5 µg/day from 0 to 50 years, 10 µg/day between 51 and 65 years, and 15 µg/day above 65 years is suggested [6]. Vitamin D3 (cholecalciferol) is mostly formed from 7-dehydrocholesterol (provitamin D3) through ultraviolet-B (UV-B) irradiation of the skin [7]. However, this amount is very often insufficient especially in winter, because the direct sun light in the north hemisphere is insufficient for adequate vitamin D production. Since different foods are a source of vitamin D, this uptake should be increased. Vitamin D3 is found only in animal sources such as fish oil and liver. The second previtamin, vitamin D2 (ergocalciferol), is produced in plants, mainly in fungi [8], and the richest sources of vitamin D may be found in wild mushrooms [4].

Mushrooms, wild, and edible or cultivated contain varying levels of vitamin D2, depending on the exposure to incidental UV light during growth [9]. Wild and edible mushrooms, e.g., *Cantharellus tubaeformis* contain 13.6–29.8 µg/100 g dry mass (DM), and large scale produced *Agaricus bisporus* in the dark 0.2 µg/100 g DM [4]. In comparison, fortified cereals only contain vitamin D2 levels of between 2.8 and 8.3 µg/100 g, whereas mushrooms produced under UV-light exposure are reported to contain above 10 µg/100 g fresh weight (approx. 100 µg/100 g DM). One portion of this mushroom has higher vitamin D levels than most foods containing vitamin D, and the vitamin D levels in this one mushroom portion are higher than the daily international requirements of vitamin D [10]. Therefore, mushrooms could be an important source for vegetarians as well as for non-vegetarians.

Previous studies reported that there is a large variance in the content of vitamin D2 in the same types of mushrooms similarly produced [11–13]. Salemi et al. described a vitamin D2 concentration of 0.004 µg/g DM in *Agaricus bisporus*, while Urbain et al. determined 0.1 µg/g DM in the same species [14]. In both studies high performance liquid chromatography (HPLC) was used for analyte separation followed by UV absorbance detection [13, 14]. Byrdwell et al. [15] reported that the extraction of vitamin D from food is a difficult and elaborate process which makes detecting vitamin D by UV absorption highly problematic. This is particularly relevant in the case of mushrooms, where the sample heterogeneity can be caused by different protein contents (10 and 30% DM) and high concentrations of carbohydrates such as structural polysaccharides like chitin, for example [16].

Other reasons for the variability of vitamin D2 concentrations, in general, are the lack of standardized reference materials as well as the lack of standardized methods [15]. Most of the published methods to date used HPLC–UV systems, but mass spectrometry (MS) is an upcoming analytical technique used in the determination of vitamin D. Mass selection can be made with a quadrupole mass filter [14] or with time of flight (Q-TOF/MS) [17]. Mass spectrometry provides extreme selectivity in the identification of complex focused analytes, but high ion suppression occurs with coeluting components leading to dramatically reduced sensitivity. The sample preparation, which is just as extensive as that applied to UV absorbance detection, is necessary, and long chromatographic run times can resolve this analytical issue.

Therefore, the aim of the present work was to develop and validate an LC–MS/MS method for the determination of vitamin D2 in different mushroom species using a relative simple sample preparation procedure and a short run time.

## Experimental

### Chemicals and reagents

Vitamin D2-d3, the deuterated internal standard (6,19, 19-d3) (1 mg, chemically pure (CP) 98%), and the pure substance vitamin D2 (1 mg; reference standard; ≥ 98% (HPLC)) were obtained from Sigma-Aldrich (Steinheim, Germany). The organic solvent methanol, hexane (both LC–MS grade), and the analytical grade chemicals, formic acid (FA) (> 99%) and dimethyl sulfoxide (DMSO), were purchased from Merck (Darmstadt, Germany). LC–MS grade water was obtained from Honeywell Riedel-de-Haën™ (Seelze, Germany).

### Examined mushroom species

*Pleurotus ostreatus* was provided from an urban organic oyster mushroom farm in Graz (Pilzkiste, Graz, Austria) using biological organic straw as the culture medium. For the first three weeks, the mushrooms grew in the dark at 25 °C followed by two weeks at 15 °C, 85–95% moisture, and illuminated by a LED lamp (365–405 nm) for 10 h per day. All the other mushroom species (*Lentinula edodes*, *brown* and *white button mushrooms*) were purchased at the “Interspar” food shop (Graz, Austria). Extractions from all four of the mushroom species (*Pleurotus ostreatus*,* Lentinula edodes*, *brown* and *white button mushrooms*) were carried for standard additional analyses.

### Sample preparation

The mushrooms were cleaned with deionized water, subsequently frozen at − 80 °C, lyophilized, powdered, and stored at room temperature in the dark. Fifty mg of each sample was weighted in Pyrex glass tubes (16 × 100 mm, Sigma-Aldrich, Steinheim, Germany). One hundred µL of the internal standard vitamin D2-d_3_ (1.00 µg/mL in methanol) were added, and the mixture was vortexed for 30 s. Then, 1.5 mL methanol was added, and after short vortexing, the samples underwent ultrasonic oscillation at 45 °C for 30 min using a SONOREX™ Digital 10 P Ultrasonic bath (BANDELIN electronic GmbH & Co. KG, Berlin, Germany). Then, 1.5 mL of methanol/water (1 + 1, v/v) was added, and after further vortex-mixing and the addition of 6 mL of hexane, the mixture underwent ultrasonic oscillation again at 45 °C for 30 min. After cooling, the samples were rotated for 10 min and centrifuged at 3000 × g for 10 min. The organic layer was transferred to a glass tube with a peaked base and blown dry with oil-free compressed air at 50 °C using a TurboVap® LV Evaporator (Biotage, Uppsala, Sweden) and re-dissolved with 150 µL of methanol in the ultrasonic bath for 5 min at 25 °C. Re-dissolved samples were centrifuged for 5 min at 3000 × g. Clear supernatants were transferred to autosampler vials with polyethylene snap-caps (La-Pha-Pack® GmbH, Langerwehe, Germany), and 50 µL was injected into the LC–MS/MS system.

### Instrumentation

Separations were carried out using an HPLC (SHIMADZU Nexera, Kyoto, Japan) coupled to a SCIEX QTRAP 6500 triple quadrupole mass spectrometer (Applied Biosystems, Framingham, MA, USA). The HPLC system was equipped with a SIL-30AC autosampler, a degasser, two binary LC-loading pumps (LC-30AD) and one eluting (quaternary) pump (LC-20ADXR), a thermostatic column compartment (CTO-20AC), a sub-controller (SUBCvp) with two rotary valves, and the system controller CBM-20A. Detection was achieved using an electrospray ionization (ESI) source in the multiple reaction monitoring (MRM) positive ionization mode. The optimized instrument settings are summarized in Table 1. Analyte concentrations were calculated using Analyst® 1.6.2 Software (SCIEX).

**Table 1:** Optimized instrument settings for vitamin D2 and the internal standard vitamin D2-d3 measurements on the SCIEX QTRAP 6500 triple quad mass spectrometer

Parameter	Operation conditions
Ion source	Turbo Spray Ion Drive
Ion spray voltage (V)	5500
Temperature of ion source (°C)	300
Curtain gas (kPa)	137.9
Nebulizer gas (gas 1) (kPa)	344.7
Heater gas (gas 2) (kPa)	344.7
Collision gas (kPa)	62.1
Scan time (sec)	0.4951
Declustering potential (V)	60
Entrance potential (V)	10
Collision energy (V)	22
Collision cell exit potential (V)	15
Vitamin D2 (m/z ratio)	397.2/107.1
Vitamin D2-d3 (m/z ratio)	400.2/162.1

*V*, volts; *°C*, degree Celsius; *psi*, pound-force per square inch; *sec*, second

### Chromatographic conditions

A dual-column heart cutting LC–MS/MS method was used for the quantification of vitamin D2. The conditions are summarized in Table 2. Fifty µL was loaded on the first column, Kinetex® 5 µm F5 100 Å, LC Column 50 × 4.6 mm (Phenomenex, Aschaffenburg, Germany) using 85% methanol as the mobile phase. At 3.00 min, when the vitamin D2 had eluted, the flow was switched to the second column (Zorbax Eclipse XDB C18; 3.0 × 100 mm; 3.5 µm, Agilent Technologies, Santa Clara, CA, USA). After the transfer of the analyte and the internal standard at 3.30 min to the second column, the valve was switched to the initial position; column one was rinsed with 85% methanol; and the cut peak was separated on the second column with a mixture of 100% methanol, 0.1% formic acid (v/v, mobile phase A), and 0.1% formic acid in MS grade water (mobile phase B). The retention time was 6.27 min for vitamin D2. Equilibration occurred with the initial conditions for 0.40 min. This type of column switching was necessary to minimize the high number of ion-suppressing and co-eluting substances. Detailed information concerning the chromatographic optimization is presented in Supplementary Table S1.

**Table 2:** Operation conditions of the ultra-high performance liquid chromatography

HPLC	Operation conditions
Loading system	
First column	Kinetex® 5 µm F5 100 Å, LC Column 50 × 4.6 mm
Pump pressure (bar)	39
Column temperature (°C)	40
Mobile phase	Methanol (85%); 0–7.50 min
Flow rate (mL min^−1^)	1.0
Injection volume (µL)	50
Eluting system	
Second column	Zorbax Eclipse XDB C18; 3.0 × 100 mm; 3.5 µm
Pump pressure (bar)	165
Column temperature (°C)	40
Mobile phase A	Methanol with 0.1% formic acid (v/v)
Mobile phase B	0.1% formic acid (v/v) in LC-grade water
Gradient	0–3.60 min (50% A and B)
	3.61–4.80 min (50% to 100% A)
	4.81–7.00 min (100% A)
	7.01–7.10 min (100% to 50% A)
	7.11–7.50 min (50% A and B)
Flow rate (mL min^−1^)	0.7

*°C*, degree Celsius

### Standard addition

For the quantitative analysis of vitamin D2 concentrations, the method of standard addition was used [18]. Therefore, a standard curve was generated with 0.0.0, 0.1, 0.2, and 0.5 µg/g DM by adding a series of different amounts of vitamin D2 (stock solution 1 µg/mL vitamin D2 in methanol) to the mushroom samples. The increasing vitamin D2 concentrations were added step-by-step to each sample, followed by the measurement of the responses resulting from the amounts of analyte added to the samples.

### Method validation

Method validation of the bioanalytical chromatographic methods, based on the recommendations published by the Food and Drug Administration (FDA), postulates the precision acceptance criteria of 15% [19, 20]. The intra- and inter-assay imprecisions of vitamin D2 were assessed by measuring five replicates of *Pleurotus ostreatus* and *Lentinula edodes* on 5 consecutive days. The limit of detection (LOD) was defined as the lowest concentration, which produced a signal at least three times higher than the average background noise. The limit of quantification (LOQ) was defined as the lowest concentration which allowed quantification with an imprecision of < 10% [20]. Both indices were determined by analyzing the serial dilutions of the lowest calibrator. Each dilution was measured five times. The recovery of vitamin D2 was determined three times in three different concentrations (0.10, 0.20, and 0.50 µg/g DM) by adding the authentic pure substance to the mushroom samples before and after extraction. The recovery was calculated as a percentage of the measured concentration in relation to the expected concentration. In order to evaluate the accuracy, the actual concentrations of vitamin D2 were determined by comparison using an external calibration curve.

An overview of different methods for the analysis of vitamin D2, the various procedures of mushroom irradiation, and their concentrations are shown in Supplementary Table S2. Despite the high analytical selectivity and sensitivity of mass spectrometry, the usage of these instruments for the determination of vitamins has not been used very often for mushrooms, so far.

## Results and discussion

The characteristics of the analytical method are presented in Table 3. The target analyte of the method is linear over the investigated concentration range with correlation coefficients (*R*^2^) of ≥ 0.999. The intra- and inter-day imprecisions were < 4.1% and < 5.8%. The LOD and LOQ were between 0.01 and 0.05 µg/g. The extraction efficiencies from the biological matrix were 91.5 ± 4.3% for vitamin D2 and 89.7 ± 2.1% for vitamin D2 d3 (Supplementary Table S1). The vitamin D2 retention time was 6.27 min, while the whole run time until the next injection was 7.50 min.

**Table 3:** Analytical performance of the optimized vitamin D2 LC–MS/MS method

Vitamin D2			
Mushroom species	*Pleurotus ostreatus*		*Lentinula edodes*
Linear range (µg/g vitamin D2)		0.1–0.5	
Correlation coefficient (r^2^)	0.999		1.000
Intra-day precision (µg/g DM)			
(Mean ± SD)	0.119 ± 0.006		0.078 ± 0.003
(CV %)	3.9		4.1
Inter-day precision (µg/g DM)			
(Mean ± SD)	0.122 ± 0.007		0.078 ± 0.004
(CV %)	5.8		5.1
LoD (µg vitamin D2/g DM)		0.01	
LoQ (µg vitamin D2/g DM)		0.05	
Recovery (%)	91.5 ± 4.3		89.7 ± 2.1

**Fig. 1:**
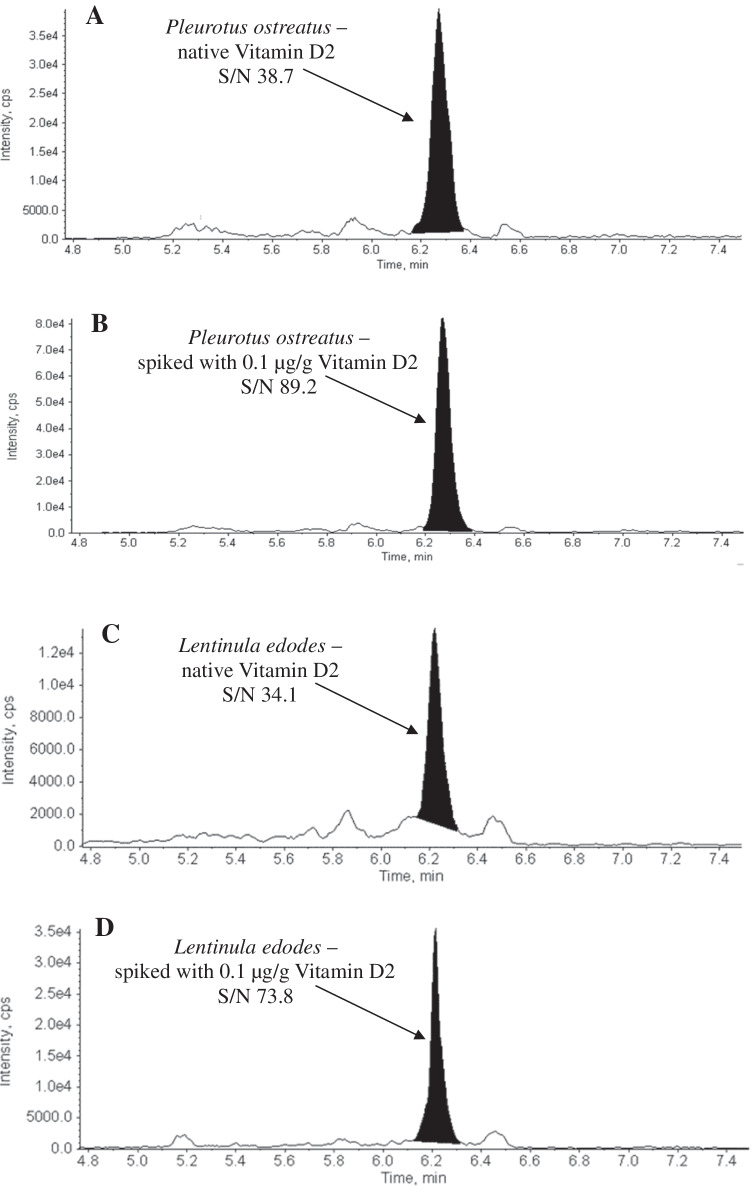
LC–MS/MS chromatograms from different mushroom species. **A** *Pleurotus ostreatus*, native (50 mg); vitamin D2 with m/z 397.2/107.1 and a retention time (RT) at 6.27 min. **B** *Pleurotus ostreatus* spiked with 0.1 µg/g vitamin D2 with a retention time (RT) at 6.27 min. **C** *Lentinula edodes*, native (50 mg); vitamin D2 with m/z 397.2/107.1 and a retention time (RT) at 6.27 min. **D** *Lentinula edodes* spiked with 0.1 µg/g vitamin D2 with a RT at 6.27 min

*m/z*, mass to charge ratio; *DM*, dry mass; *SD*, standard deviation; *CV*, coefficient of variation; *LoD*, limit of detection; *LoQ*, limit of quantification;

The vitamin D2 concentrations of the mushroom samples were calculated using a standard addition analysis. Chromatograms from both mushroom species without vitamin D2 and spiked with 0.1 µg/g of vitamin D2 are shown in Fig. 1. Furthermore, calibration curves were generated for the four mushroom species in order to ensure accuracy (Supplementary Figure S1). A mean vitamin D2 concentration of 0.122 ± 0.007 µg/g DM was determined in *Pleurotus ostreatus*. *Lentinula edodes* samples contained a mean vitamin D2 concentration of 0.078 ± 0.004 µg/g DM (Table 3). *Brown* and *white button mushrooms* had mean vitamin D2 concentrations of 0.099 ± 0.007 µg/g DM and 0.073 ± 0.005 µg/g DM.

Similar low vitamin D2 concentrations were published by Mattila et al. [21]. They reported vitamin D2 concentrations ranging from 0.026 to 3.72 µg/g DM in differently cultivated mushroom species [21]. This study does not describe the UV irradiation of the samples. Jasinghe and Perera measured vitamin D2 concentrations of 6.05 ± 0.07 µg/g DM in *Lentinula edodes* and 4.4 ± 0.08 µg/g DM [22] in *Pleurotus ostreatus* purchased from a supermarket in Singapore. After 2 h of UV irradiation, the concentrations increased to 22.8 ± 1.07 and 45.1 ± 3.07 µg/g DM.

Another study by Gyôrfi et al. described a vitamin D2 content of 0.67 µg/g DM in cultivated pre-harvest UV-irradiated *Pleurotus ostreatus* samples [11]. Huang et al. found up to 12.7 ± 1.54 µg/g DM in *Pleurotus ostreatus* and 17.16 ± 0.47 µg/g DM in *Lentinula edodes* samples before UV irradiation. After a 60-min light treatment, the concentrations reached 229 µg/g DM. [23]. The *Pleurotus* *ostreatus* samples examined were indeed irradiated with LED light (365–405 nm). However, the wavelength did not provide an optimal range for vitamin D2 formation. Román-Hidalgo et al. reported that UV-B radiation (280–315 nm) is the most effective wavelength in stimulating the production of vitamin D2 in mushrooms [17]. This could be one explanation for the low level of vitamin D in our *Pleurotus* *ostreatus* samples, where higher values were expected. As for the other samples (*Lentinula edodes*, *brown* and *white button* *mushrooms*), there were no information about the UV irradiation nor about the sunlight exposure and the growing conditions. Therefore, the present results cannot be compared with the published values of 0.005 µg/g DM [24] or even 0.004 µg/g DM in untreated mushrooms [13]. The studies mentioned above applied HPLCUV methods to measure vitamin D concentrations [11, 13, 21, 22]. Moreover, high vitamin D levels have also been published using mass spectrometry methods. Sławinska et al. reported vitamin D2 amounts in untreated *Pleurotus ostreatus* species of 56.6 ± 0.82, of 29.3 ± 2.24 in *Lentinula edodes* and of 13.1 ± 1.6 µg/g DM in *white button mushrooms* using an UHPLC system coupled to a triple quadrupole with electrospray ionization [25].

What could the potential reasons for these discrepancies be? At the beginning of the twenty-first century, it had already been postulated that vitamin D2 is totally absent in cultivated mushrooms (≤ 0.1 µg/ g DM) due to the lack of sunlight exposure and the harvest in the dark [26, 27]. In contrast to the cultivated species without UV irradiation, wild mushrooms contain higher vitamin D2 concentrations [4, 28]. However, cultivated mushrooms with UV irradiation have markedly higher vitamin D2 content than in mushrooms, which grow in the wood. Various studies (listed in Supplementary Table S2) investigated UV-irritation using different wavelengths and exposure times and numerous mushroom species with differently prepared pieces, which demonstrated highly variable amounts of vitamin D2 levels [12, 23, 25, 29].

There are a few analytical reasons why such different vitamin D2 results have been obtained. Numerous organic solvents are used for several clean-up steps (e.g., saponification, the extraction, and purification of the extract) to avoid the matrix effects of the mushroom ingredients [21, 23, 24]. Up to now, the determination of vitamin D2 has been mainly performed using the reversed-phase HPLC with UV absorbance detection ranging from 250 to 280 nm [30, 31]. Mass spectrometry methods certainly fit better for this approach and were used by few authors [17, 25, 32, 33]. This analytical technique has the advantage of simultaneously quantifying multiple compounds with high sensitivity and selectivity [34]. However, ion suppression and the occurrence of interferences also require elaborate sample preparation steps and long separation run times.

Therefore, the present study aimed at developing a LC–MS/MS method with a relative simple extraction procedure, selective chromatographic separation, and a short run time.

First, a one-column method was tested with a selective sample procedure including saponification and different extraction steps to generate a clean, interference-free extract. Although a complex sample preparation method was used, the ion suppression of the coeluted substances was so high that only a 10% response of added pure substance in the mushroom extract was found compared to the same amount of analyte injected in the mobile phase. In this form, the method was unsuitable for vitamin D2 measurement in mushroom samples. Based on these findings, the strategy was changed to a simplified sample procedure. The mushroom samples were sonicated with methanol/water (1 + 1, v/v), extracted with hexane, and were successfully analyzed using the two-dimensional method. The attempt to increase the vitamin D concentration by multiple extractions provided only slight increases in yield. The increase in yield was always associated with a higher proportion of interfering factors. The recovery of the target analyte is at a reasonable level (> 90%). It does not seem necessary to increase it because a labeled internal standard has been inserted into the matrix. This internal standard distributes in the matrix like the endogenous analyte and can compensate the extraction losses very well.

It is a common practice to re-dissolve the sample in the mobile phase to achieve optimum peak shape and to avoid the deposition of substances when the methanol content of the mobile is reduced. To bring the whole amount of analyte in solution of the dried samples, 100% methanol was used. Injection of samples containing higher methanol concentration than the mobile phase often results in problems. Switching to the second column with post-focus chromatographic separation gave a good peak shape, so we did not further optimize the injection solvent and first mobile phase. The rinsing of the first column was done with 100% methanol so that possible substance depositions on the first column are removed again.

To the best of our knowledge, this is the first method for vitamin D measurement in mushrooms using two different columns in a heart cutting setting in combination with a triple quadrupole system to analyze vitamin D content with a low impairment in ionization. Our LC–MS/MS method has an interference free stable LOQ of 0.05 µg/g DM. This LOQ is adjusted to the concentration present in the mushrooms and can still be lowered considerably by increasing the amount of tissue used, by lowering the dissolution volume, or by increasing the injection volume.

Our study harbors some limitations that should be mentioned. In this developed two-dimensional LC–MS/MS method, vitamin D2 from Sigma-Aldrich was used, which is a certified reference material (CRM). However, no standard reference controls were available for vitamin D2 analysis in mushrooms. Such certified materials are necessary to generate accuracy concentrations. Additionally, the development of standardized reference methods would be very important. Furthermore, it must be mentioned that only cultivated mushrooms were determined in this work; only information on the growing conditions of one species was available.

In conclusion, the well-optimized, sensitive LC–MS/MS method with a simple sample preparation and a short run time presented here demonstrates that vitamin D2 detection in different mushroom species is possible even without UV-treatment. In order to get accurate information about mushrooms as a rich source of vitamin D2, further studies especially with UV irradiation are necessary. They may improve our current knowledge about vitamin D2 levels in mushrooms. The method presented here is a reliable tool for vitamin D2 quantification in mushrooms.

## Supplementary Information

Below is the link to the electronic supplementary material.

Supplementary file1 (DOCX 114 KB)
