# Supplementary material for: Development of a two-dimensional liquid chromatography-tandem mass-spectrometry method for the determination of vitamin D2 in mushrooms
Source: Anal Bioanal Chem. 2022 Aug 19;414(26):7565–72. doi: 10.1007/s00216-022-04281-3 (PMC9587085; doi:10.1007/s00216-022-04281-3)
Supplement: Supplementary file 1 — (DOCX 114 KB) [file 216_2022_4281_MOESM1_ESM.docx]

**Supplementary**

**Supplementary Table S1.** Method optimization of the sample preparation and chromatographic separation

Method information RT (min) Recovery (%) Ion suppression (%)

Vitamin D2 / Vitamin D2-d_3_

**Sample preparation changes**

Sample preparation according to Huang et al. (10) 13.77 / 13.76 77.3/ 83.3 93.9/92.5

Sample preparation without DMSO 13.77 / 13.76 86.7/87.2 75.9/81.6

**Chromatographic changes**

From one to two column system 13.77 / 13.76 87.1/79.3 24.5/22.6

Mobile phase: 95 % methanol (instead of 100 %) 10.11 / 10.11 82.1/84.5 21.2/23.7

Mobile phase: 85 % methanol (instead of 90 %) 8.11/8.14 95.0/85.1 15.6/19.9

Faster gradient (Eluting system, Table 2) 6.27/6.27 92.5/91.9 12.2/9.9

RT = retention time.

**Supplementary Table S2.** Overview of the effects of UV-irradiation on contents of vitamin D2 in different mushroom species

Author Species UV-irradiation Intensity Method Vitamin D2 (µg/g DW)

(Source) (Hours / Temperature) (Before/after irradiation)

Jasinghe et al [25] *Lentinula edodes* UV-A 2 h, 27 °C, 25.2 kJ/m^2^ HPLC-UV - /45.1 ± 3.1

Teichmann et al [30] *Agaricus bisporus* UV-C (254nm) 30 min, 25 °C, 94.7 J/cm^2^ HPLC-UV 0.07/5.32

1 h, 25 °C, 189.5 J/cm^2^ - /7.24

2 h, 25 °C, 379.0 J/cm^2^ - /10.14

*Agaricus bisporus* UV-A (366 nm) 30 min, 25 °C, 94.7 J/cm^2^ HPLC-UV 0.07/0.09

1 h, 25 °C, 189.5 J/cm^2^ - /0.11

2 h, 25 °C, 379.0 J/cm^2^ /0.15

Roberts et al [20] *Agaricus bisporus* UV-B 18 min, 5 kJ/m^2^ HPLC-MS 0.01/3.8 ± 0.2

36 min, 10 kJ/m^2 -^ /4.5 ± 0.2

54 min, 15 kJ/m^2 -^ /7.3 ± 0.3

Koyyalamudi et al [21] *Agaricus bisporus* UV-B/C 11.5 kJ/m^2^ per pulse HPLC-APCI-MS/MS

3 pulses - /8.9 ± 0.9

6 pulses - /15.9 ± 1.7

9 pulses - /20.9 ± 2.2

Gyôrfi et al [9] *Pleurotus ostreatus* UV-B (312 nm) 15 min HPLC-UV 0.67/0.70

Gyôrfi et al [9] *Pleurotus ostreatus* UV-B (312 nm) 90 min HPLC-UV - /3.69

UV-C (254 nm) 15 min HPLC-UV 0.67/1.26

90 min - /0.70

Huang et al [10] *Lentinula edodes* 280 – 360 nm 2 h, 25 °C HPLC-UV 0.35 ± 0.05/15.1 ± 1.55

*King oyster* 280 – 360 nm 2 h, 25°C 1.56 ± 0.17/28.7 ± 0.96

*Golden oyster* 280 – 360 nm 2 h, 25°C 3.93 ± 0.44/209 ± 6.08

*Oyster* 280 – 360 nm 2 h, 25 °C 0.83 ± 0.06/69.0 ± 1.96

*Pink oyster* 280 – 360 nm 2 h, 25 °C 2.13 ± 0.21/93.3 ± 11.6

Sławinska et al [13] *Agaricus bisporus* Hot-air dried, UV-B 5 min, 69 mJ/cm^2^ UHPLC-MS/MS - /21.5 ± 1.1

10 min, 69 mJ/cm^2 -^ /34.8 ± 0.5

15 min, 69 mJ/cm^2^ - /53.3 ± 1.1

20 min, 69 mJ/cm^2^ - /71.2 ± 0.6

30 min, 69 mJ/cm^2^ - /81.2 ± 1.9

*Agaricus bisporus* Freeze dried, UV-B 5 min, 69 mJ/cm^2^ - /42.1 ± 0.9

10 min, 69 mJ/cm^2^ - /79.9 ± 2.5

15 min, 69 mJ/cm^2^ - /91.1 ± 2.2

20 min, 69 mJ/cm^2^ - /100 ± 2.2

30 min, 69 mJ/cm^2^ - /119 ± 1.4

*Pleurotus ostreatus* Hot-air dried, UVB 5 min, 69 mJ/cm^2^  - /5.5 ± 0.4

10 min, 69 mJ/cm^2^ - /23.5 ± 1.9

15 min, 69 mJ/cm^2^ - /28.1 ± 1.5

Sławińska et al [13] *Pleurotus ostreatus* Hot-air dried, UVB 20 min, 69 mJ/cm^2^ UHPLC-MS/MS - /40.0 ± 1.5

30 min, 69 mJ/cm^2^ - /31.8 ± 1.9

*Pleurotus ostreatus* Freeze dried, UV-B 5 min, 69 mJ/cm^2^ - /23.8 ± 1.2

10 min, 69 mJ/cm^2^ - /40.7 ± 1.0

15 min, 69 mJ/cm^2^ - /59.0 ± 1.5

20 min, 69 mJ/cm^2^ - /45.7 ± 1.4

*Pleurotus ostreatus* Freeze dried, UV-B 30 min, 69 mJ/cm^2^ - /34.6 ± 0.9

*Lentinula edodes* Hot-air dried, UV-B 5 min, 69 mJ/cm^2^ - /7.8 ± 1.1

10 min, 69 mJ/cm^2^ - /20.0 ± 1.2

15 min, 69 mJ/cm^2^ - /26.7 ± 1.1

20 min, 69 mJ/cm^2^ - /31.3 ± 1.8

30 min, 69 mJ/cm^2^ - /41.6 ± 1.2

*Lentinula edodes* Hot-air dried, UV-B 5 min, 69 mJ/cm^2^ - /11.2 ± 1.9

10 min, 69 mJ/cm^2^ - /18.9 ± 1.4

15 min, 69 mJ/cm^2^ - /27.5 ± 1.3

20 min, 69 mJ/cm^2^ - /41.2 ± 1.5

30 min, 69 mJ/cm^2^ - /59.9 ± 1.4

Huang et al [28] *Pleurotus ostreatus* UV-C 10 min, 0.25 J/cm^2^ HPLC-UV 12.67 ± 1.54/123

30 min, 0.25 J/cm^2^ - /201

60 min, 0.25 J/cm^2^ - /230

Urbain et al [12] *Agaricus bisporus* Sun exposure 60 min HPLC-DAD 0.1/3.9 ± 0.8

Urbain et al [12] *Agaricus bisporus* UV-B 15 min, 0.13 J/cm^2^ HPLC-DAD - /2.2 ± 0.5

UV-B 0.53 J/cm^2^ - /67.1 ± 9.9

UV-B 2.01 J/cm^2^ - /101.5 ± 4.3

UV-B 1.5 and 1.8 J/cm^2^ - /55 - 60

Nölle et al [14] *Agaricus bisporus* UV-B 20 min, 1.5 J/cm^2^ HPLC-UV

Whole (UV followed by freeze drying) - /44 ± 3

Slice (UV followed by freeze drying) - /406 ± 59

Slice (UV followed by hot-air drying) - /384 ± 16

Slice (freeze dried followed by UV) - /395 ± 5

Slice (freeze dried powder followed by UV) - /177 ± 14

Keflie et al [32] *Pleurotus ostreatus* Sun exposure 30 min - /28.5 ± 8.5

1 h - /36.6 ± 8.7

3 h - /73.7 ± 12.9

8 h - /64.2 ± 9.3

16 h - /88.1 ± 3.1

Salemi et al [11] *Agaricus bisporus* UV-B 15 min, 27 °C, 12.5 kJ/m^2^ HPLC-UV 0.004/1.87

30 min, 27 °C, 12.5 kJ/m^2^ - /3.28

45 min, 27 °C, 12.5 kJ/m^2^ - /3.50

60 min, 27 °C, 12.5 kJ/m^2^ - /3.77

90 min, 27 °C, 12.5 kJ/m^2^ - /5.16

120 min, 27 °C, 12.5 kJ/m^2^ - /3.6

Román-Hidalgo et al [15] *Agaricus bisporus* UV-C (254 nm) 15 min, 23 °C UPLC-Q-TOF/MS - /1.9 ± 1.0

UV-B (302 nm) - /23.0 ± 7.9

**A**


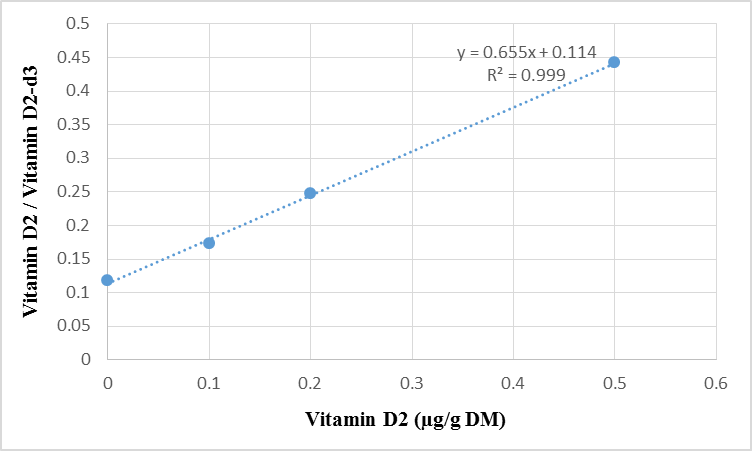


**B**


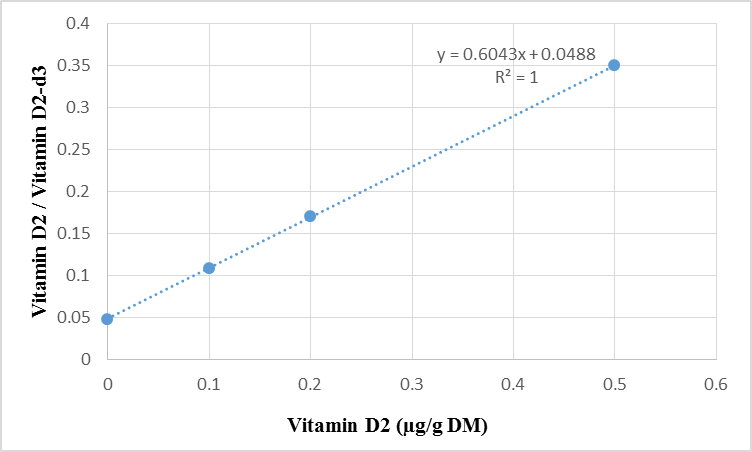


**C**


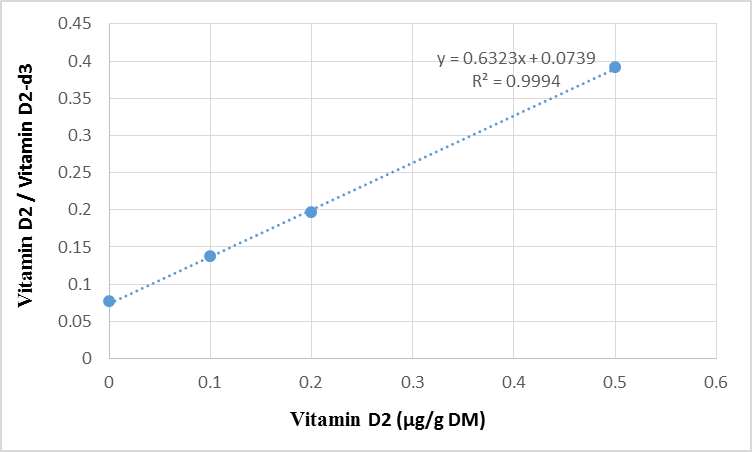


**D**


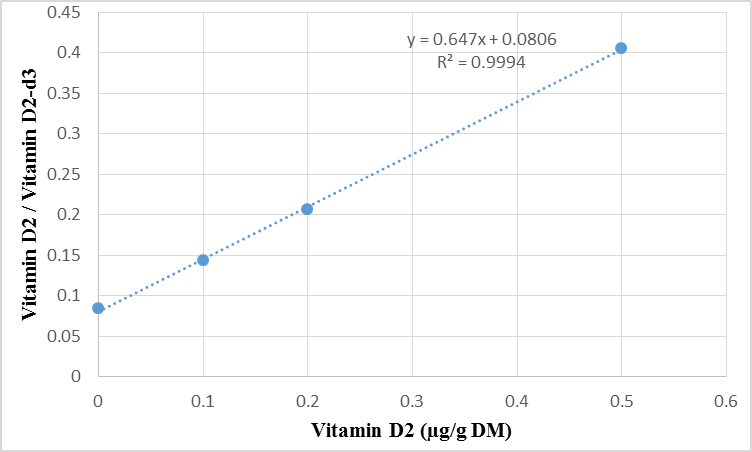


**Supplementary Figure S1**

**Supplementary Figure Legend S1**

Calibration curves of *Pleurotus ostreatus,* *Lentinula edodes, Brown* and *White Champignons*. (A) *Pleurotus ostreatus* spiked with 0.1, 0.2 and 0.5 µg vitamin D2. (B) *Lentinula edodes* spiked with 0.1, 0.2 and 0.5 µg vitamin D2. (C) *Brown champignon* spiked with 0.1, 0.2 and 0.5 µg vitamin D2. (D) *White champignon* spiked with 0.1, 0.2 and 0.5 µg vitamin D2.
